# Rehabilitation technologies and interventions for individuals with spinal cord injury: translational potential of current trends

**DOI:** 10.1186/s12984-018-0386-7

**Published:** 2018-05-16

**Authors:** Kristin E. Musselman, Meeral Shah, José Zariffa

**Affiliations:** 1 Toronto Rehabiltiation Institute-University Health Network, Toronto, Canada; 2 Department of Physical Therapy, Faculty of Medicine, University of Toronto, Toronto, Canada; 3 Rehabilitation Sciences Institute, Faculty of Medicine, University of Toronto, Toronto, Canada; 4 Institute of Biomaterials and Biomedical Engineering, University of Toronto, Toronto, Canada

**Keywords:** Spinal cord injury, Rehabilitation, Technology, Clinical implementation, Environmental scan

## Abstract

In the past, neurorehabilitation for individuals with neurological damage, such as spinal cord injury (SCI), was focused on learning compensatory movements to regain function. Presently, the focus of neurorehabilitation has shifted to functional neurorecovery, or the restoration of function through repetitive movement training of the affected limbs. Technologies, such as robotic devices and electrical stimulation, are being developed to facilitate repetitive motor training; however, their implementation into mainstream clinical practice has not been realized. In this commentary, we examined how current SCI rehabilitation research aligns with the potential for clinical implementation. We completed an environmental scan of studies in progress that investigate a physical intervention promoting functional neurorecovery. We identified emerging interventions among the SCI population, and evaluated the strengths and gaps of the current direction of SCI rehabilitation research. Seventy-three study postings were retrieved through website and database searching. Study objectives, outcome measures, participant characteristics and the mode(s) of intervention being studied were extracted from the postings. The FAME (**F**easibility, **A**ppropriateness, **M**eaningfulness, **E**ffectiveness, **E**conomic **E**vidence) Framework was used to evaluate the strengths and gaps of the research with respect to likelihood of clinical implementation. Strengths included aspects of **F**easibility, as the research was practical, aspects of **A**ppropriateness as the research aligned with current scientific literature on motor learning, and **E**ffectiveness, as all trials aimed to evaluate the effect of an intervention on a clinical outcome. Aspects of **F**easibility were also identified as a gap; with two thirds of the studies examining emerging technologies, the likelihood of successful clinical implementation was questionable. As the interventions being studied may not align with the preferences of clinicians and priorities of patients, the **A**ppropriateness of these interventions for the current health care environment was questioned. **M**eaningfulness and **E**conomic **E**vidence were also identified as gaps since few studies included measures reflecting the perceptions of the participants or economic factors, respectively. The identified gaps will likely impede the clinical uptake of many of the interventions currently being studied. Future research may lessen these gaps through a staged approach to the consideration of the FAME elements as novel interventions and technologies are developed, evaluated and implemented.

**Electronic supplementary material:**

The online version of this article (10.1186/s12984-018-0386-7) contains supplementary material, which is available to authorized users.

## Background

The past three decades have seen a shift in the focus of neurorehabilitation from the use of compensatory approaches to enable function toward an emphasis on functional neurorecovery, or promoting the restoration of function through use of the affected limbs. The shift from compensation to neurorecovery has been most apparent in the rehabilitation of individuals with incomplete spinal cord injury (SCI) [1]. For example, 30 years ago the rehabilitation of walking for those with incomplete SCI consisted of learning to use assistive devices. As basic research concerning the spinal control of locomotion was translated to humans, the potential for these individuals to *recover* independent walking ability was revealed. Through repetitive exposure to weight-supported stepping on a treadmill, spinal cats regained some walking function [2]. These discoveries, and their subsequent translation to humans with incomplete SCI [3], resulted in a paradigm shift from compensation to neurorecovery.

Our understanding of the potential for neuroplasticity following a SCI has contributed to the development of intensive physical interventions that aim to promote neurorecovery through repetitive movement training [4]. To facilitate the intensive training process, therapeutic technologies have been developed, such as exoskeletons, functional electrical stimulation (FES), and robotic rehabilitation devices. While clinicians have an interest in incorporating technology that may augment neurorecovery into practice, the actual uptake of technology in neurorehabilitation has been low [5–7].

As consumers and providers of rehabilitative interventions, clinicians must consider client preferences, scientific evidence, previous clinical experience and available resources (e.g. equipment, time, staffing) when choosing an intervention. The FAME (**F**easibility, **A**ppropriateness, **M**eaningfulness, **E**ffectiveness and **E**conomic **E**vidence) framework was developed to assist clinicians with the implementation of evidence-based practices [8, 9]. The framework outlines five elements to consider when deciding whether or not to implement an intervention or technology.

1. *Feasibility*: whether the intervention is practical (i.e. concerned with the actual doing of something rather than theory) and practicable (i.e. the ability to successfully implement into practice) given the cultural, physical and financial context.
2. *Appropriateness*: whether the intervention fits within a therapeutic scenario and the current health care context.
3. *Meaningfulness*: whether the intervention and outcomes matter to the target population, setting and culture. Meaningfulness relates to the personal experience, perceptions, values, thoughts, and beliefs of each individual patient.
4. *Effectiveness*: whether the intervention achieves the intended effect, which may be a clinical or health service outcome.
5. *Economic Evidence*: whether the cost-to-benefit ratio supports implementation of the intervention.

While the intended users of the FAME framework are clinicians, knowledge translation and implementation are processes shared amongst the research and clinical communities. Ideally researchers also consider the FAME elements when designing novel interventions and research studies for neurorehabilitation. Interventions and technologies satisfying the FAME elements may be adopted more readily by clinicians and hence, make more tangible impacts on patient outcomes and the neurorehabilitation field.

Given the shift in the focus of SCI rehabilitation from compensation to neurorecovery, and the development of technology to support this direction, we wished to consider how SCI rehabilitation research aligns with the potential for clinical implementation. To do this we completed an environmental scan of current SCI rehabilitation research to: 1) Identify emerging physical interventions that aim to promote neurorecovery among the SCI population, and 2) Evaluate the strengths and gaps of the current direction of SCI rehabilitation research using the FAME framework as a guide for evaluation.

## Methods

An environmental scan of clinical trials for the SCI population was completed from June 8–July 5 2017. Environmental scans are often performed to examine a field as a whole in order to provide evidence of its current direction, to raise awareness of issues or gaps, and to plan future initiatives and strategies [10].

### Search strategies

Sources of information included databases or websites that list ongoing research studies for SCI. Due to the time lag between the conduct and publication of research studies, we examined research currently being conducted and not yet published. Nine websites listing SCI research studies were accessed: ClinicalTrials.gov, Craig H. Neilsen Foundation, Spinal Cord Outcomes Partnership Endeavor, International Collaboration On Repair Discoveries, Wings for Life, Unite 2 Fight Paralysis, and Rick Hansen Institute, whose website also directed us to the websites for Spinal Cord Injury Ontario and Spinal Cord Injury Alberta. The research studies listed on these websites were reviewed by two authors (MS, KEM) to identify studies adhering to the following inclusion/exclusion criteria:

Inclusion criteria:

- Participants included individuals with SCI (traumatic or non-traumatic cause) aged ≥16 years at any time since injury (i.e. < 1 year post-injury or acute/sub-acute, and ≥ 1 year post-injury or chronic).
- The primary intervention(s) being studied was a physical intervention (not a surgical, pharmacological or cell-based intervention) that aimed to promote neurorecovery.
- Study status was either active but not yet recruiting, recruiting, enrolling, or data collection complete but not yet published.

Exclusion criteria:

- Studies with an unknown status that had not been updated in > 2 years, or for which published results were found.
- Insufficient study information (> 50% of study details were missing, see Data Extraction below).
- Cross-sectional studies (i.e. studies involving a single exposure to a physical intervention as this was unlikely to induce neurorecovery).

All study postings were reviewed on each website, with the exception of Clinicaltrials.gov where a search was performed. The search criteria used reflected the above inclusion and exclusion criteria.

### Data extraction and synthesis

Information about each included study was extracted from the website and compiled into a data extraction table by one author (MS). Information gathered included: source of study posting, study status, location of study, study objectives, outcome measures, and injury characteristics of the targeted participants, such as neurological levels of injury and time since injury. We also documented whether the study included an evaluation of the economic feasibility, participants’ perspectives (e.g. qualitative interviews) and an assessment of the clinical utility (i.e. ease of use in a clinical environment) of the intervention(s) being examined.

Each study was classified by the type or mode of physical intervention used. Due to our focus on interventions that promote neurorecovery, we anticipated that the majority of interventions would be activity-based therapies (ABT). With ABT neuroplasticity is driven by repetitive neuromuscular activation below the level of spinal injury, typically achieved through intensive, task-specific movement practice. Some modes were anticipated in advance and identified a priori; for example, robotics, virtual reality, electrical stimulation, and repetitive movement training without emerging technology (e.g. treadmill or overground gait training). Interventions using electrical stimulation were further divided into three groups according to the site of stimulation: brain, spinal cord or peripheral nervous system (i.e. FES). The target function of the intervention was also noted (i.e. upper extremity function, lower extremity function, and/or trunk function).

### Evaluation of strengths and gaps

The strengths and gaps of current SCI rehabilitation research were identified through consideration of the five FAME elements and the environmental scan results. Feasibility was divided into separate evaluations of whether the studied intervention was practical and practicable, and Appropriateness was divided into four sub-elements: institution, clinician, patient and scientific evidence. The authors determined, through consensus, whether each element or sub-element reflected a strength or a gap. Answering ‘yes’ to the following guiding questions suggested that the element was a strength.

1. *Feasibility:* Practical: Does the study involve application of an intervention to a target population? Practicable: Does the study involve an intervention that is clinically feasible? For example, an intervention requiring specialized equipment and/or knowledge or training would be considered less clinically feasible. Is a measure of clinical utility included in the study?
2. *Appropriateness:* Institution: Do hospitals/rehabilitation centers commonly have the resources (e.g. space, manpower, finances) required to administer the intervention? Clinician: Is the intervention likely to meet the needs of clinicians? For example, does it reflect real-world tasks, measure performance, and encourage variability in movement patterns while simultaneously preventing practice of compensatory movement strategies [6]? Patient: Is the intervention likely to meet the needs of individuals with SCI participating in rehabilitation, given their priorities [11] and stage of recovery? Scientific Evidence: Does the intervention align with principles of motor learning and neuroplasticity [12]? For example, does the intervention involve motor practice that is task-specific and intensive (i.e. many repetitions), and elicits voluntary participation from the patient?
3. *Meaningfulness:* Does the study include documentation of the participants’ perspectives on the intervention and/or its intended outcome? Meaningfulness may be assessed through qualitative methods, such as semi-structured interviews or focus groups, or through goal attainment scaling. Structured questionnaires may also probe meaningfulness, albeit without the depth of understanding provided by qualitative methodologies.
4. *Effectiveness*: Does the study include evaluation of the effect of the intervention?
5. *Economic Evidence*: Does the study include evaluation of economic factors?

## Results

### Environmental scan results

A total of 1614 study postings were found. After de-duplicating and screening for inclusion, 73 studies were included. Sixty-nine of the 73 studies were returned in the ClinicalTrials.gov database. This database involves a review process that provides standardization for study description; hence, the quality of study postings was high for the majority of included postings. Most studies (*n* = 49, 67%) were actively recruiting participants (Table 1). The majority of research studies were being conducted in North America (*n* = 54, 74%) or Europe (*n* = 15, 21%). Two studies were being completed in Thailand, one in Israel and one in China. The bias of inclusion toward the western world likely reflects the sources of information accessed for the environmental scan.

**Table 1:** Study and participant characteristics

	Number of studies (% of sample (*n* = 73))
Study status	
Not yet recruiting	7 (10%)
Actively recruiting	49 (67%)
Ongoing study, no longer recruiting	10 (14%)
Data collection complete	3 (4%)
Not specified^a^	4 (5%)
Participant Characteristics	
Injury Severity:	
Incomplete	33 (45%)
Complete	3 (4%)
Incomplete & complete	9 (12%)
Not specified	28 (39%)
Time Since Injury^b^	
Chronic	27 (37%)
Sub-acute	7 (10%)
Chronic & sub-acute	28 (38%)
Not specified	11 (15%)

^a^Study status not reported, but published study results were not found

^b^Chronic = ≥ 1 year post-injury; sub-acute = < 1 year post-injury

Given our focus on neurorecovery, it was not surprising that the sub-group of SCI most commonly targeted was individuals with incomplete SCI (*n* = 33, 45%) (Table 1). Participants with chronic injuries (37%, *n* = 27) were more commonly targeted than those with sub-acute SCI (*n* = 7, 10%).

Electrical stimulation was the mode of intervention being studied in 20 studies (27%) (Table 2). Various applications of brain stimulation were being investigated, as was epidural stimulation of the spinal cord, and surface or implanted FES systems for the upper or lower limbs and/or trunk. Sixteen studies (22%) were examining repetitive movement training without the use of emerging technology, such as overground gait training, balance exercises, upper limb exercises and dance/yoga, to name a few (Table 2). ABT with robotic devices were being investigated in 15 studies (21%); with the majority employing lower extremity exoskeletons or the Lokomat®. Six study postings described research on the effects of intermittent hypoxia on voluntary movement and function, primarily of the lower extremity. Five research studies were incorporating a form of virtual reality into the ABT. Nine studies (12%) employed a combination of two forms of electrical stimulation, such as brain stimulation paired with peripheral nerve stimulation (i.e. paired associative stimulation), or a combination of stimulation with movement practice using a robotic device.

**Table 2:** Intervention characteristics

	Number of studies (% of total (*n* = 73))
Mode	
Electrical stimulation	20 (27%)
Brain	8 (11%)
tDCS	2 (3%)
rTMS	4 (5%)
DBS	2 (3%)
Spinal Cord	4 (5%)
Epidural	4 (5%)
PNS (i.e. FES)	8 (11%)
Surface FES for UE	4 (5%)
Surface FES for UE and LE (cycling)	1 (1%)
Implanted FES for LE	2 (3%)
Implanted FES for trunk	1 (1%)
Repetitive Movement Training^a^	16 (22%)
Overground gait training	5 (7%)
“Locomotor training”^b^	2 (3%)
BWSTT	1 (1%)
TT in aquatic environment	1 (1%)
Cycling	1 (1%)
Dance/yoga	1 (1%)
Balance exercises	1 (1%)
Weight-bearing exercises^c^	2 (3%)
UE exercises	2 (3%)
Robotics	15 (21%)
LE exoskeleton	9 (12%)
Lokomat	3 (4%)
UE robotic device (1 brain-controlled)	2 (3%)
LE & UE robotic device	1 (1%)
Combination	9 (12%)
Two types of stimulation (i.e. brain + SC or FES)	6 (8%)
Robotics + stimulation (i.e. brain or SCI)	3 (4%)
Intermittent Hypoxia	6 (8%)
LE function	5 (7%)
UE function	1 (1%)
Virtual Reality	5 (7%)
Walking/LE function	3 (4%)
UE function & trunk (virtual sailing)	1 (1%)
Not specified	1 (1%)
Whole-body vibration (LE)	1 (1%)
Low-level laser therapy^d^	1 (1%)
Target Function	
LE	46 (63%)
UE	15 (22%)
Trunk (seated balance)	1 (1%)
LE & UE	10 (13%)
UE & trunk	1 (1%)

^a^Without technology or therapeutic assistive devices

^b^Details of locomotor training not specified

^c^Sit-to-stand exercises in one study

^d^Aim is to affect sensory and motor function. *tDCS* = transcranial direct current stimulation; *rTMS* = repetitive transcranial magnetic stimulation; *DBS* = deep brain stimulation; *PNS* = peripheral nerve stimulation; *FES* = functional electrical stimulation; *LE* = lower extremity; *UE* = upper extremity; *BWSTT* = body weight-supported treadmill training; *TT* = treadmill training; *SC* = spinal cord; *SCI* = spinal cord injury

### Strengths and gaps

The FAME elements of feasibility (practical), appropriateness (scientific evidence), and effectiveness were identified as strengths of current SCI rehabilitation research, while the remaining FAME elements were deemed to be gaps (see Table 3).

1. *Feasibility*: Current SCI rehabilitation research is practical; it is concerned with the application of interventions. The interventions being researched are less practicable. Two thirds of the research involved electrical stimulation, robotics, virtual reality, or a combination of these technologies, all of which require specialized equipment and knowledge. Ten research postings (14%) listed a measure of clinical utility among the study outcomes.
2. *Appropriateness*: Institution: Financial, staffing and space constraints likely limit the appropriateness of the interventions currently being studied. The expertise required to operate and to repair emerging technology, such as robotic devices and brain stimulation, is typically not found amongst the neurorehabilitation team.

**Table 3:** Strengths and gaps

FAME Element	
Feasibility	
Practical	+
Practicable	–
Appropriateness	
Institution	–
Clinician	–
Patient	–
Scientific Evidence	+
Meaningfulness	–
Effectiveness	+
Economic Evidence	–

**+** indicates a strength; **−** indicates a gap

Clinician: Although many of the interventions attempt to mimic real-world tasks, such as overground walking, the extent to which the interventions emphasize the quality of movement (i.e. minimization of compensatory movement strategies) is not clear.

Patient: Lower extremity function, including walking, was most commonly targeted by the studies (*n* = 46, 63%). However, regaining arm and hand function is the top priority for individuals with incomplete tetraplegia [11], who represent 45% of the SCI population [13]. In addition, little research (*n* = 2, 3%) was focused on improving trunk function, which is a prerequisite for effective movement of the upper limb. Furthermore, SCI rehabilitation research is focused on chronic SCI, most likely to eliminate the effects of natural recovery. Inclusion of participants with sub-acute SCI would be more appropriate for the therapeutic context (i.e. inpatient and outpatient rehabilitation), and aligns with the window of opportunity for neuroplasticity [4, 12].

Scientific Evidence: Many of the interventions being studied were designed with the principles of motor learning and neuroplasticity in mind. The majority of interventions involved repetitive, task-specific movement training. The exceptions were studies (*n* = 8, 10%) evaluating the effects of intermittent hypoxia, whole body vibration and low-level laser.

3) *Meaningfulness*: Only five studies (7%) documented the participants’ perspectives on the effects of an intervention (i.e. Canadian Occupational Performance Measure (*n* = 3) or a semi-structured interview (*n* = 2)). An additional six (8%) included a questionnaire about participants’ opinions on the device or intervention being tested. About one third of studies included a measure of quality of life; however, quality of life and meaningfulness may not equate.
4) *Effectiveness*: The majority of studies included measures of body structure and function or activity according to the International Classification of Functioning, Disability and Health (ICF) [14]. Measures of gait (*n* = 41, 56%), balance/postural control (*n* = 25, 34%), upper extremity function (*n* = 19, 26%), independence (n = 19, 26%), strength (*n* = 17, 23%), and sensorimotor function (e.g. the International Standards for Neurological Classification of SCI (ISNCSCI) exam, motor evoked potentials) (*n* = 11, 15%) were included.
5) *Economic Evidence*: Only two of the 73 studies (3%) mentioned evaluating one or more economic factors. One study planned to document the number of staff required during two modes of locomotor training, as well as other economic factors that were not specified in the posting. The second study planned to evaluate whether locomotor training affects the use of a personal care attendant and/or in-home nursing care.

## Discussion

We completed an environmental scan to identify 73 active studies researching physical interventions that promote neurorecovery among adults with SCI. Emerging interventions included repetitive movement practice supplemented with technology, such as robotic devices, virtual reality and electrical stimulation of the central or peripheral nervous systems. What is the likelihood that these interventions can be successfully implemented into clinical practice? The clinical uptake of these technologies into SCI rehabilitation has received little study to-date; however, previous work in stroke rehabilitation would suggest the likelihood is not high. FES, robotics and Wii or Kinect systems are infrequently used by clinicians working with individuals who have experienced a stroke [5–7]. Numerous barriers to the clinical implementation of rehabilitative technology exist. For example, a lack of knowledge about the technology combined with little or no allocated time to learn [6, 7]. The technology may be perceived too time-consuming to set-up and/or administer, detracting from a patient’s allocated therapy time [6, 7]. Technologies may be perceived to disrupt clinician-patient interactions [6]; for example, physical therapists prefer to use their hands to facilitate movement rather than FES [7]. There are financial barriers as well. The cost and maintenance of some technologies may exceed what a clinical environment can afford. Identifying and addressing barriers early in the development and study of interventions is crucial for the research to clinical translation [15].

The Effectiveness element of the FAME framework was considered a strength of current SCI rehabilitation research. The evaluation of the effects of an intervention can occur under controlled conditions, such as in a laboratory setting; referred to as an efficacy trial [16]. Alternatively an intervention may be studied under ‘real-world’ conditions, as would be the case if the study setting was a hospital and/or with a heterogeneous group of patients; this is an effectiveness trial [16]. While the FAME Framework does not distinguish between efficacy and effectiveness, we believe it is important to acknowledge that these two types of trials address different research questions, with efficacy trials evaluating whether an intervention works and how large the effect is, and effectiveness trials evaluating whether the intervention’s efficacy persists in clinical practice [16]. We suspect that the majority of studies included in the environmental scan were efficacy trials. Moving forward, the study of SCI rehabilitation interventions should include efficacy trials followed by evaluations of effectiveness if efficacy was achieved, as this process is likely to facilitate the identification of barriers and implementation in clinical environments.

From the environmental scan it seemed that few studies planned to evaluate relevant economic factors; however, it is possible that a separate economic analysis was planned, but not reported. Further, the two studies that included an economic aspect specified resource utilization (i.e. staffing) as the focus. It may be that more detailed and lengthy economic analyses, such as the number of quality-adjusted-life-years (QALY) afforded by an intervention [15], are not considered until the effectiveness of an intervention is established. It has also been suggested that unfavorable findings from economic analyses can stall the research of novel technologies that only benefit a small group of people, such as those with SCI [15]. Hence, there may be justification to perform economic analyses at a later stage in the research process.

**Fig. 1:**
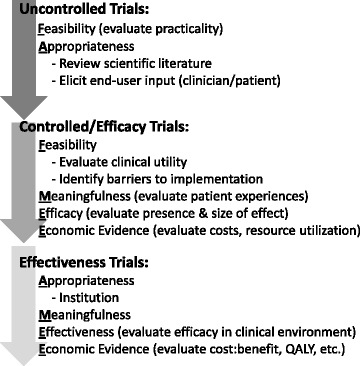
A staged approach to the incorporation of FAME elements into the research of novel interventions and technologies. QALY = quality-adjusted-life-years

In sum, there are gaps in current SCI rehabilitation research that will likely impede the clinical implementation of many of the interventions under investigation. Future research should aim to lessen these gaps through consideration of the FAME Framework during the development, evaluation and implementation of novel rehabilitation interventions or technologies. We suggest a staged approach to the consideration of FAME elements (Fig. 1). For example, in the early stage of uncontrolled trials, researchers may consider the **F**easibility (practicality) and begin an evaluation of **A**ppropriateness by considering the face validity of the intervention. This may involve scanning relevant literature and eliciting input from end-users. Incorporating feedback from clinicians into the design of interventions, or adopting user-centered designs, may lessen the gap between the interventions being studied and the therapeutic context [15]. Following this early stage, controlled or efficacy trials are encouraged to collect data concerning **M**eaningfulness, efficacy (**E**ffectiveness) and **E**conomic factors (i.e. costs and resource utilization), in addition to the ongoing consideration of practicality (**F**easibility) and **A**ppropriateness. It would also be an appropriate time to consider the clinical utility of the intervention and anticipate the barriers to its implementation – i.e., how practicable the intervention is (**F**easibility). Finally, the **E**ffectiveness of the intervention may be evaluated, as well as consideration of the **A**ppropriateness of the intervention at the level of the health care institution. An **E**conomic analysis that considers cost:benefit or QALY may also be completed. By considering the FAME Framework throughout the research process, the successful translation of novel interventions or technologies from research to clinical practice may be realized.

While the FAME elements provide a framework to comprehensively evaluate the translational potential of new rehabilitation interventions, it is important to consider the elements of the model together rather than in isolation. For example, if an intervention is found to be highly effective, a consensus may be reached that some modifications to clinical processes are warranted in order to incorporate the new technique. These modifications might in turn entail a reevaluation of feasibility considerations.

## Conclusion

In current SCI rehabilitation research, the use of technology to augment physical interventions was found to be common; however, the feasibility, appropriateness, meaningfulness and economic impact of these new therapies require greater consideration. Ongoing dialogues between clinicians and researchers can ensure that new technologies and ideas are vetted rigorously while informing changes to practice that will maximize outcomes for individuals with SCI.

## Additional file

Additional file 1: Supplementary Info - Data Extraction Table JNER. (XLSX 91 kb)

## Abbreviations

ABT: Activity-based therapies
BWSTT: Body weight-supported treadmill training
DBS: Deep brain stimulation
FAME: Feasibility, Acceptability, Meaningfulness, Effectiveness, Economic Evidence
FES: Functional electrical stimulation
LE: Lower extremity
PNS: Peripheral nerve stimulation
QALY: Quality-adjusted-life-years
rTMS: repetitive transcranial magnetic stimulation
SC: Spinal cord
SCI: Spinal cord injury
tDCS: transcranial direct current stimulation
TT: Treadmill training
UE: Upper extremity
